# The Dual Role of RUNX1 in Inflammation-Driven Age-Related Diseases: From Molecular Mechanisms to Clinical Translation

**DOI:** 10.3390/biomedicines13122999

**Published:** 2025-12-07

**Authors:** Kexin Chen, Si Wang

**Affiliations:** Department of Cardiology, West China Hospital, Sichuan University, Chengdu 610041, China; chenkexin@wchscu.cn

**Keywords:** RUNX1, inflammaging, cellular senescence, epigenetic remodeling, age-related diseases

## Abstract

Age-related diseases such as cardiovascular disorders, neurodegeneration, and metabolic syndrome share a unifying pathological signature—persistent low-grade inflammation or “inflammaging”. Among the transcriptional regulators that orchestrate this process, RUNX1 has emerged as a pivotal molecular hub linking inflammation, cellular senescence, and tissue dysfunction. Traditionally recognized for its role in hematopoietic lineage specification, RUNX1 is now known to exert context-dependent regulatory functions across diverse organ systems. Its activation in aged tissues is driven by convergent pro-inflammatory and stress-related pathways—including NF-κB, MAPK, JAK/STAT, and oxidative signaling—that reinforce RUNX1 transcriptional activity through epigenetic reprogramming and chromatin remodeling. Sustained RUNX1 upregulation contributes to cellular senescence, fibrotic remodeling, and regenerative blockade, forming a self-perpetuating cycle of “inflammation amplification–functional decline”. In the cardiovascular, nervous, and hematopoietic systems, aberrant RUNX1 activation underlies fibrosis, neuroinflammation, and clonal hematopoiesis, respectively, establishing RUNX1 as a shared driver of age-associated pathology. The isoform-specific and temporally dynamic regulation of RUNX1 underpins its dual pro- and anti-inflammatory roles, highlighting its translational potential as both a biomarker and therapeutic target. A range of emerging intervention strategies has demonstrated promising capacity to precisely modulate RUNX1 activity. Collectively, these advances position RUNX1 at the intersection of inflammation, epigenetic instability, and tissue degeneration, opening new avenues for targeted intervention in inflammaging and age-related diseases.

## 1. Introduction

With the accelerating pace of global population aging, age-related diseases have emerged as a major public health challenge. These conditions—including cardiovascular disease, neurodegenerative disorders, metabolic syndrome, sarcopenia and cancer—share a common pathological feature: the persistent presence of low-grade chronic inflammation. This phenomenon, termed “inflammaging”, reflects a progressive imbalance in immune regulation, disruption of tissue homeostasis, and maladaptive remodeling during ageing [1,2,3]. Inflammation not only contributes to disease onset and progression but also influences therapeutic responsiveness and clinical outcomes. Therefore, elucidating the molecular mechanisms underlying inflammatory regulation is essential for developing targeted interventions in age-related diseases.

Within the inflammatory regulatory network, transcription factors serve as pivotal mediators that bridge immune homeostasis and pathological inflammation by modulating the expression of inflammation-related genes. *RUNX1* (Runt-related transcription factor 1), a central member of the Runt family, has traditionally been recognized as a master regulator of hematopoietic development and lineage specification, with its dysregulation closely linked to various hematologic malignancies [4,5]. More recently, RUNX1 has garnered attention beyond the hematopoietic system, where it exhibits bidirectional roles—both pro-inflammatory and anti-inflammatory—across diverse inflammatory conditions. These effects are highly context-dependent, shaped by cell type, differentiation status, and microenvironmental cues [6]. This conditional responsiveness underscores the biological complexity of RUNX1 and highlights its potential as a diagnostic marker and therapeutic target in inflammation-associated diseases.

Notably, *RUNX1* is closely associated with aging and plays a pivotal role in regulating cell fate and maintaining tissue homeostasis across multiple organ systems [7,8,9]. Previous studies have demonstrated that *RUNX1* expression undergoes dynamic changes during aging, and its upregulation—triggered by various intrinsic and extrinsic factors—can promote cell cycle arrest, DNA damage responses, and transcription of pro-inflammatory and senescence-associated genes, thereby inducing cellular senescence and sustaining low-grade chronic inflammation [10,11]. RUNX1 also enhances cellular sensitivity to oxidative stress by activating aging-related signaling pathways, accelerating tissue functional decline [12]. In hematopoietic stem cells and the cardiovascular system, aberrant RUNX1 activation contributes to stem cell exhaustion and the maintenance of a pro-inflammatory microenvironment, further linking RUNX1 to age-associated tissue remodeling.

Collectively, these findings delineate a biological landscape in which RUNX1 serves as a central node connecting inflammation, aging, and multi-organ pathology. Despite growing evidence of its multifaceted roles in inflammatory responses, the mechanisms by which RUNX1 operates remain highly context-dependent, influenced by cell type, temporal dynamics, and disease-specific conditions. A unified theoretical framework to integrate RUNX1’s dynamic functions in age-related diseases is still lacking. This review aims to systematically examine the molecular mechanisms by which RUNX1 regulates inflammation, with a particular focus on its dual pro- and anti-inflammatory roles in aging-relevant cell types and disease models. We hope to provide conceptual and translational insights into the “RUNX1–inflammation–aging” axis, paving the way for precision-targeted interventions in age-associated disorders.

## 2. Molecular Characteristics and Expression Profile of RUNX1

RUNX1 is the central member of the RUNX transcription factor family. Its protein structure comprises a highly conserved Runt homology domain (RHD) and a C-terminal transcriptional regulatory domain. The RHD mediates specific DNA binding and forms a heterodimer with core-binding factor β (CBFβ), which enhances the stability and transcriptional activity of RUNX1. The protein also contains N-terminal sequences determined by distinct promoter usage, along with C-terminal activation and repression domains that provide the structural basis for recruiting co-activators or co-repressors and mediating downstream transcriptional effects [13,14].

**Figure 1:**
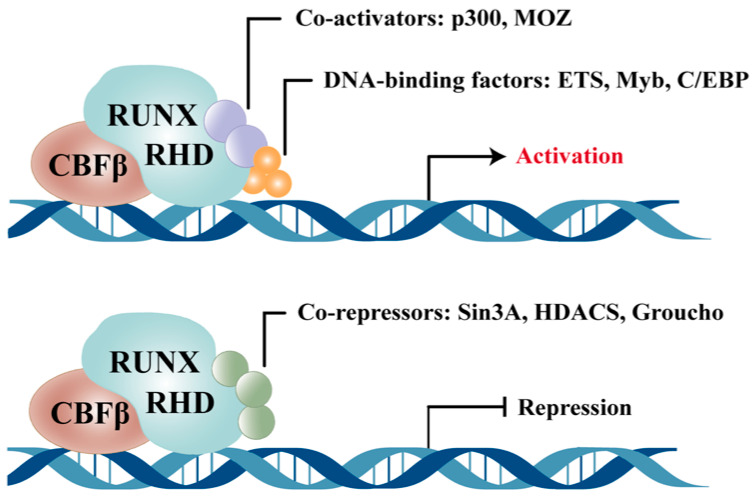
Schematic illustration of the dual transcriptional roles of RUNX1 as both an activator and a repressor. RHD: Runt homology domain; RUNX: Runt-related transcription factor; CBFβ: core-binding factor β.

The *RUNX1* gene is subject to complex alternative splicing regulation, primarily driven by two promoters: the distal P1 and proximal P2. These generate three major isoforms—*RUNX1a*, *RUNX1b*, and *RUNX1c*. *RUNX1a* lacks the C-terminal regulatory domain and functions as a transcriptional repressor, whereas *RUNX1b* and *RUNX1c* retain the full-length structure and exhibit transcriptional activation capacity. These isoforms display distinct expression patterns across cell types and developmental stages, suggesting potential functional divergence or even antagonism in inflammatory regulation [15,16] (Figure 1).

**Figure 2:**
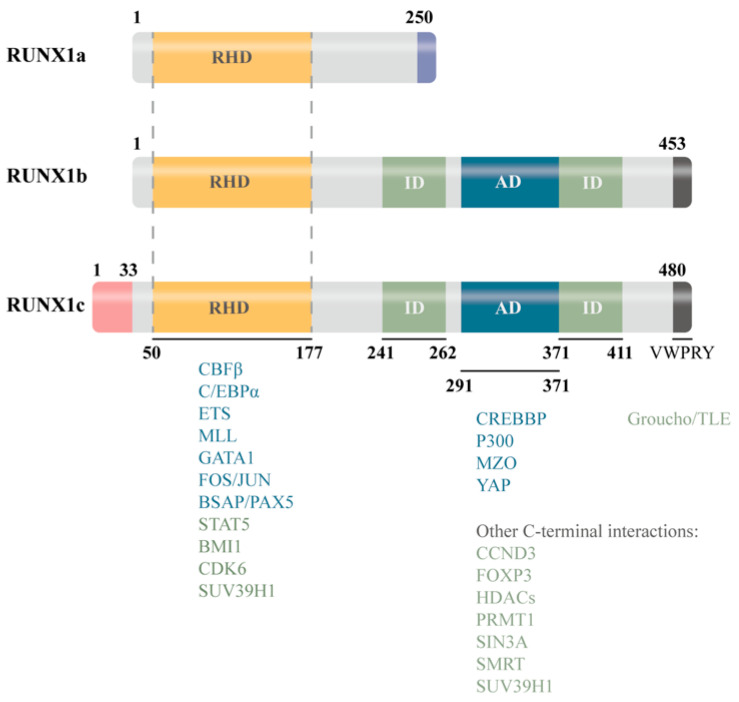
Overview of the transcriptional profiles and structural features of the three *RUNX1* isoforms. BMI1: BMI1 Proto-Oncogene, Polycomb Ring Finger; BSAP/PAX5: B-cell Specific Activator Protein/Paired Box 5; CBFb: Core-Binding Factor beta; CCND3: Cyclin D3; CDK6: Cyclin-Dependent Kinase 6; C/EBPa: CCAAT/Enhancer-Binding Protein alpha; CREBBP: CREB Binding Protein; ETS: E26 Transformation-Specific family; FOS/JUN: FBJ Murine Osteosarcoma Viral Oncogene/Jun Proto-Oncogene, AP-1 complex; FOXP3: Forkhead Box P3; GATA1: GATA Binding Protein 1; Groucho/TLE: Transducin-Like Enhancer of Split family; HDACs: Histone Deacetylases; MLL: Mixed-Lineage Leukemia, KMT2A; MZO: Magnesium Zinc Oxide; P300: E1A Binding Protein p300; PRMT1: Protein Arginine Methyltransferase 1; SIN3A: SIN3 Transcription Regulator Family Member A; SMRT: Silencing Mediator for Retinoid and Thyroid Receptors; STAT5: Signal Transducer and Activator of Transcription 5; SUV39H1: Suppressor of Variegation 3–9 Homolog 1; YAP: Yes-Associated Protein.

In terms of cellular expression, *RUNX1* is broadly expressed throughout the hematopoietic system and serves as a central regulator of lineage specification. Single-cell and histological analyses have demonstrated its widespread expression in myeloid compartments—including monocytes/macrophages and neutrophils—as well as its essential role in megakaryocyte and platelet lineages [17,18,19]. Increasing evidence also supports the presence of *RUNX1* in several non-hematopoietic tissues, where it contributes to growth and development and homeostatic processes [20,21,22]. This broad yet stratified expression profile underpins the cell-type specificity of *RUNX1* in immune and inflammatory responses (Figure 2, Table 1).

**Figure 3:**
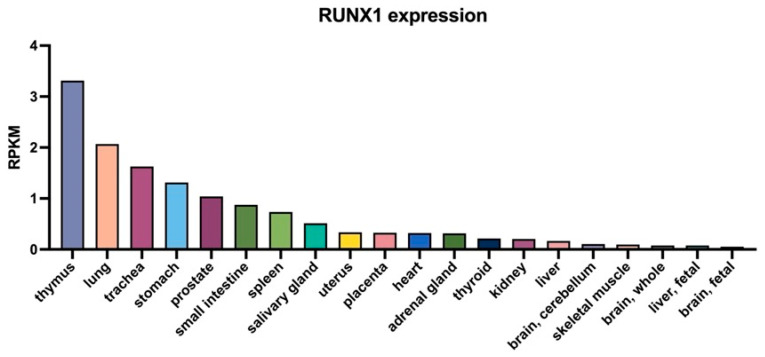
Tissue-specific expression profile of *RUNX1* in human organs. The bar graph illustrates the expression levels of the *RUNX1* gene (RUNX family transcription factor 1) across 20 distinct human tissues, based on RNA sequencing data. Expression is quantified as RPKM (Reads Per Kilobase of transcript, per Million mapped reads), providing a normalized measure of transcript abundance. Notably, *RUNX1* exhibited the highest expression in lymphoid and hematopoietic-related tissues such as spleen and lymph node, with moderate levels in ovary and other epithelial organs. These data suggest a potential role for RUNX1 in immune regulation and tissue-specific transcriptional activity. Expression data were retrieved from the NCBI Gene Expression database (BioProject: PRJNA280600).

The functional diversity of RUNX1 is shaped by its modular protein structure, isoform heterogeneity, layered expression profile, and interactions with key transcriptional regulators. RUNX1 does not act in isolation; rather, its transcriptional output is determined by context-specific molecular interactions. The heterodimeric complex with CBFβ forms the core DNA-binding unit. Its interaction with PU.1 is particularly critical in myeloid gene regulation, where RUNX1 modulates PU.1’s recruitment of co-repressors or co-activators, thereby influencing target gene activity and lineage commitment [23,24,25,26]. RUNX1 also engages with the NF-κB pathway at multiple levels: it can directly bind the p50 subunit and interfere with the IKK complex, modulating NF-κB signaling strength and downstream inflammatory gene transcription [27,28,29]. Additionally, RUNX1 recruits or excludes histone-modifying enzymes such as Histone Deacetylases and other epigenetic regulators to exert long-term control over target gene expression [30,31,32]. These interaction networks help explain the divergent transcriptional responses of RUNX1 under varying cellular and stimulatory conditions, resulting in either pro-inflammatory or anti-inflammatory phenotypes. Thus, the domain architecture, isoform diversity, expression landscape, and transcriptional partnerships of RUNX1 collectively define its molecular plasticity and context-dependent roles in inflammatory regulation. To further highlight the functional differences of RUNX1 across distinct organ systems, we have summarized its roles in the heart, brain, and bone marrow in a comparative table, enabling readers to more intuitively grasp its multidimensional pathological significance (Table 2, Figure 3).

## 3. RUNX1 in Age-Associated Inflammation

To better interpret the role of RUNX1 in age-associated inflammation, it is essential to first examine its tissue-specific expression across human organs (Figure 3).

### 3.1. Inflammatory Signaling-Driven Activation of RUNX1

In aged tissues, the persistent presence of chronic low-grade inflammation (inflammaging), coupled with oxidative stress, represents a major driver of sustained *RUNX1* upregulation. This transcriptional activation is orchestrated through a multilayered regulatory framework involving signal transduction cascades, transcription factor interactions, and chromatin remodeling. RUNX1 activation is not only an early event in tissue remodeling during age-related disease progression, but also serves as a transcriptional hub across diverse pathological contexts.

Multiple studies have shown that canonical pro-inflammatory cytokines—including IL-1β, TNF-α, IL-6 and IFN-γ—are consistently elevated in the aging microenvironment. These signals not only perpetuate chronic inflammation but also directly modulate *RUNX1* transcriptional activity via several converging pathways. At the molecular level, the NF-κB, MAPK and JAK/STAT axes are considered key conduits for transmitting inflammatory signals to the *RUNX1* promoter. NF-κB can bind directly to upstream cis-regulatory elements of the *RUNX1* promoter, enhancing transcriptional initiation. Meanwhile, STAT3 and p38 MAPK promote RUNX1 phosphorylation, facilitating its nuclear localization and DNA-binding affinity, thereby amplifying its transcriptional output. Notably, elevated *RUNX1* expression can further reinforce inflammatory signaling, contributing to a feed-forward loop of cytokine induction [33,34,35,36,37,38,39].

Building on the activation of RUNX1 by exogenous inflammatory stimuli, aging cells also exhibit a range of intrinsic stress responses that contribute to its sustained upregulation. Oxidative stress, a hallmark of the aging microenvironment, promotes RUNX1 phosphorylation and nuclear accumulation via ROS-mediated activation of the p38 MAPK and JNK pathways [40,41]. This enhances RUNX1’s transcriptional activity on cell cycle inhibitory genes, thereby reinforcing proliferative arrest and the maintenance of senescent phenotypes. Concurrently, DNA damage responses remain chronically active in aged cells, and elevated RUNX1 expression may further exacerbate oxidative stress and genomic instability, suggesting that RUNX1 functions not only as a downstream effector of stress signaling but also as an amplifier within pathological feedback circuits [28,42,43].

Mitochondrial dysfunction—characterized by membrane potential collapse and accumulation of metabolic intermediates—can activate the Nrf2 and AMPK pathways, indirectly modulating *RUNX1* expression and activity. *RUNX1* upregulation, in turn, suppresses PI3K–Akt signaling, leading to reduced mitochondrial membrane potential, impaired ATP production and elevated ROS levels. These findings indicate that RUNX1 plays a central role in regulating mitochondrial homeostasis and contributes to metabolic reprogramming and functional decline in aging cells [23,44,45,46].

Epigenetic remodeling further supports *RUNX1* overexpression by altering chromatin accessibility. Mutations within its transcriptional activation domain can indirectly affect DNA methylation patterns and disrupt megakaryocyte differentiation, highlighting RUNX1’s cooperative role in epigenetic programming [47,48]. Single-cell integrative analyses have revealed strong correlations between RUNX1 activity and histone modifications—including H3K27ac, H3K4me3, H3K4me1, H3K36me3 and H3K27me3—suggesting that stress signals may sustain RUNX1 activation through epigenetic amplification mechanisms [49].

Importantly, these intrinsic mechanisms do not operate in isolation but are tightly coupled with external inflammatory cues. For instance, Toll-like receptors activated by pathogen-associated or sterile inflammatory stimuli can engage the MyD88–NF-κB axis to further enhance *RUNX1* expression [50]. Similarly, senescence-associated secretory phenotype (SASP) factors such as CXCL1, IL-8, and MMPs can feed back to activate RUNX1, establishing a self-reinforcing inflammatory loop [51,52,53]. Collectively, these findings position RUNX1 not merely as a passive responder to inflammation and stress, but as an active participant in sustaining inflammatory persistence, stabilizing senescent phenotypes and reshaping cell fate progression in aging tissues.

### 3.2. RUNX1-Mediated Tissue Dysfunction and Regenerative Impairment

In age-related pathological contexts, sustained activation of RUNX1 not only maintains a pro-inflammatory microenvironment but also disrupts tissue homeostasis and regenerative programs, emerging as a key driver of functional decline across multiple organ systems. Its roles in the cardiovascular, nervous, and hematopoietic systems are particularly prominent, reflecting a systemic manifestation of inflammaging.

In aging cardiac tissue, *RUNX1* contributes to both inflammation amplification and regenerative suppression, acting as a central mediator of myocardial remodeling. It is upregulated in cardiac fibroblasts and identified as a key gene in myocardial hypertrophy and fibrosis. Mechanistically, RUNX1 promotes fibroblast activation through several pathways: by stimulating TGF-β signaling, cooperating with CBX4 to induce fibrosis-related gene expression, and recruiting histone acetyltransferase P300 to epigenetically activate periostin transcription. These processes drive myofibroblast differentiation, extracellular matrix deposition and pressure overload-induced cardiac fibrosis, ultimately exacerbating ventricular remodeling and contractile dysfunction. RUNX1 also integrates into the MBNL1-regulated fibrotic signaling module, directly enhancing αSMA expression. Simultaneously, RUNX1 suppresses the proliferation and differentiation of cardiac progenitor cells, limiting post-injury repair capacity [45,54,55,56,57,58]. This dual role—as both a fibrosis inducer and regeneration inhibitor—establishes a pathological loop of “inflammation–fibrosis–regeneration blockade”, wherein persistent *RUNX1* expression drives structural remodeling and impairs tissue recovery. In atherosclerosis, RUNX1 has been implicated in reprogramming neutrophils toward a pro-inflammatory phenotype and promoting clonal expansion and phenotypic switching of vascular smooth muscle cells. It is also activated in macrophages and other myeloid cells, driving inflammatory gene expression and accelerating plaque formation and disease progression [52,59,60,61,62].

In aging-related neurological disorders, RUNX1 functions as a transcriptional regulator of early immune responses, inflammation amplification, and regenerative inhibition. Its aberrant activation is closely linked to neuroinflammation and axonal regeneration failure. Elevated *RUNX1* expression in astrocytes and microglia modulates inflammatory signaling and intercellular communication, promoting cytokine release and exacerbating neuronal damage and synaptic degeneration. RUNX1 cooperates with STAT3 and TGF-β pathways to drive injury-associated transcriptional programs, contributing to neuroinflammation and dementia pathology. It also activates the RUNX1–TOLLIP–TLR3 axis in astrocytes, inducing inflammatory responses and neuronal injury. In neurons, RUNX1 suppresses axon growth-related genes and neurotrophic signaling, impairing plasticity and repair capacity, and may even trigger cell cycle dysregulation and programmed cell death [53,63,64]. Furthermore, RUNX1 regulates chromatin accessibility and transcriptional network remodeling, restricting neural stem cell differentiation and contributing to neuronal functional decline [65,66]. This closed-loop mechanism of “inflammation amplification–regeneration blockade–functional deterioration” positions RUNX1 not only as an inflammatory regulator but also as a maintenance module of aging programs, offering a novel transcriptional target for intervention in neurodegenerative and age-related neurological dysfunction.

In the aging hematopoietic system, persistent RUNX1 activation profoundly reshapes lineage trajectories, giving rise to an “inflammatory hematopoiesis” phenotype. RUNX1 is essential for adult hematopoietic stability and leukemia suppression, and its dysfunction leads to impaired stem cell differentiation, myeloid malignancies, and aberrant lineage specification [17,67,68]. This skewed differentiation compromises immune diversity and adaptability, while sustained release of inflammatory cells exacerbates bone marrow inflammation, forming a vicious cycle of “inflammation–hematopoietic imbalance–microenvironmental deterioration”. Importantly, RUNX1 interacts with epigenetic regulators that frequently acquire somatic mutations in aged individuals, contributing to clonal hematopoiesis of indeterminate potential and progression toward bone marrow malignancies [69,70,71]. These mechanisms underscore RUNX1’s central role in age-associated hematopoietic dysfunction and its potential pathogenic relevance in myelodysplastic syndromes and acute myeloid leukemia. RUNX1 thus represents a critical node linking chronic inflammation, epigenetic instability, and hematopoietic aging.

Notably, RUNX1 also reinforces the senescence-associated secretory phenotype (SASP) in aging tissues by co-activating SASP factor expression. This sustains autocrine inflammatory signaling and promotes paracrine-mediated functional decline in neighboring cells. Even in the absence of external stimuli, senescent cells with elevated *RUNX1* continuously secrete inflammatory mediators, establishing a self-perpetuating “inflammatory memory” that underlies chronic inflammation, tumorigenesis, and organ degeneration [12,72,73]. RUNX1 can also slow osteoarthritis progression in aging mice, suggesting it as a potential therapeutic target [74,75,76]. RUNX1’s role in maintaining this inflammatory feedback loop highlights its potential as a therapeutic target in age-related tissue dysfunction.

In summary, RUNX1 serves as a central regulatory node in the inflammatory landscape of aging, acting both as a sensor of immune activation and an amplifier of pathological remodeling. Driven by convergent inflammatory cytokines and intrinsic stress signals, and reinforced by chromatin remodeling, RUNX1 sustains long-term transcriptional activity that links transient stress to chronic tissue dysfunction. By integrating inflammatory signaling with epigenetic regulation, RUNX1 orchestrates fibrosis, regenerative blockade, and lineage skewing across cardiovascular, neural, and hematopoietic systems, thereby coupling cellular senescence to systemic aging. Its dual role as both downstream effector and upstream driver establishes a self-perpetuating circuit of “inflammation–epigenetic instability–regeneration failure”, which underlies inflammatory memory and age-related decline. These insights position RUNX1 as a molecular nexus connecting inflammaging, epigenetic drift, and organ failure, and highlight its potential as a transcriptional target for interventions aimed at reversing or delaying age-associated diseases.

### 3.3. Clinical Relevance and Translational Potential of RUNX1

As a central transcriptional regulator of inflammation, RUNX1 is increasingly transitioning from mechanistic investigation to translational application. Its multifaceted roles in immune lineage specification, cytokine expression, tissue repair, and fibrotic remodeling have been implicated in a wide range of diseases, where it holds diagnostic, prognostic, and therapeutic value [77,78,79] (in vitro, mouse models, human data). With growing insights into the mechanisms of inflammaging, RUNX1 has emerged as a key contributor to age-related pathologies, particularly through its roles in sustaining chronic inflammation, promoting maladaptive tissue remodeling, and impairing regenerative capacity [80,81,82] (mouse models, human data). These findings suggest that RUNX1 is not only a marker of cellular aging but also a potential target for modulating age-associated disease trajectories. However, significant limitations remain in current RUNX1-targeted therapies. These include challenges in isoform selectivity, off-target effects, and difficulties in achieving precise temporal control. Further research is needed to optimize these approaches and overcome these hurdles. Future efforts must focus on delineating isoform-specific functions and temporal expression dynamics, enabling the development of cell type- and ageing stage-specific intervention models. Such strategies will be essential for advancing RUNX1-targeted therapies into clinical validation and for supporting precision medicine approaches in chronic age-related disorders.

### 3.4. Isoform-Specific Functional Dissection: The Foundation for Precision Targeting

*RUNX1* generates multiple isoforms (*RUNX1a*/*b*/*c*) through alternative promoter usage (P1/P2) and splicing, each exhibiting distinct expression patterns across cell lineages, inflammatory states, and developmental stages. *RUNX1a*, which lacks the C-terminal regulatory domain, often functions as a dominant-negative isoform that promotes hematopoietic stem cell proliferation [15,83] (mouse models, in vitro). *RUNX1b*, transcribed from the proximal promoter, undergoes m^6^A methylation, which modulates eNOS expression and endothelial function, thereby contributing to blood pressure regulation [84] (in vitro, human endothelial cells). *RUNX1c*, driven by the distal promoter, along with *RUNX1b*, differentially regulates gene expression in megakaryocytes and platelets, influencing transcriptional programs associated with acute cardiovascular events [16].

In inflammatory contexts, isoform-specific activity also shapes immune cell responses—for example, by modulating NF-κB signaling to enhance pro-inflammatory outputs or by differentially regulating fibrosis-related genes during tissue remodeling [85,86]. However, isoform-specific functions remain underexplored, and a better understanding of these differences will be crucial for precise targeting. To enable precise targeting, future studies should integrate isoform-specific RNA-seq, CUT&RUN/CUT&Tag, ChIP-seq, translatomics, and protein interactome profiling to construct a comprehensive “isoform–binding site–downstream pathway” map.

### 3.5. Intervention Models and Temporal Control: Identifying the Therapeutic Window

*RUNX1* function is highly time-dependent, with its expression dynamics across the initiation, propagation, and resolution phases of inflammation determining both the safety and efficacy of therapeutic intervention. Developing models with lineage specificity and temporal control is therefore critical. Tools such as inducible Cre systems, degron-based protein degradation tags, and conditional knockout/knock-in mouse models—combined with humanized organoids and single-cell lineage tracing—can enable high-resolution monitoring of *RUNX1* activity [87,88] (mouse models, human organoids). These platforms will help define reversible “therapeutic windows” and critical “risk thresholds” during disease progression. For instance, early inhibition of *RUNX1* following myocardial infarction has demonstrated a time-sensitive therapeutic benefit, supporting the existence of a defined intervention window [23] (mouse models). Such findings underscore the potential of RUNX1-targeted strategies in both acute inflammation and tissue repair phases.

### 3.6. Drug Development: From Transcriptional Inhibition to Precision Delivery

Due to its nuclear localization and lack of enzymatic activity, RUNX1 has long been considered “undruggable”. However, recent advances have yielded promising strategies targeting its key interaction interfaces like small-molecule inhibitors: Ro5-3335, a well-characterized compound that disrupts *RUNX1*–CBFβ binding, has been widely used in preclinical studies to attenuate *RUNX1* transcriptional activity [89,90]. Targeted protein degradation can also be a way. Proteolysis-targeting chimeras leverage the ubiquitin–proteasome system to selectively degrade RUNX1, offering high specificity and efficacy in degrading oncogenic transcription factors [91]. RNA-based silencing is a mature method for therapy. siRNA encapsulated in immunolipid nanoparticles enables cell-specific silencing of *RUNX1* in vivo, effectively reducing adhesion molecule expression and inflammatory signaling [92]. Some studies have also employed intraocular delivery of mRNA encoding the dominant-negative *RUNX1* inhibitor (RUNX1-Trap), encapsulated within polymer–lipid complexes or lipid nanoparticles [93]. These approaches provide both pharmacological and delivery-system foundations for the clinical translation of RUNX1-targeted therapies.

### 3.7. Clinical Trials and Disease Stratification: RUNX1 as a Dual-Purpose Biomarker

RUNX1 is now entering clinical evaluation as both a therapeutic target and a biomarker. A Phase Ib trial initiated by the U.S. National Cancer Institute (NCT06090669) is assessing the effect of imatinib on restoring *RUNX1* function in patients with germline *RUNX1* mutations. Another NIH-sponsored study (NCT03854318) is longitudinally tracking disease progression and phenotypic outcomes in individuals with germline *RUNX1* variants, supporting its utility in early screening and risk stratification. Additionally, multicenter cohort studies has linked *RUNX1* mutations to lower remission rates and higher relapse risk, underscoring its prognostic relevance and potential to inform treatment decisions and response prediction [94,95,96].

**Table 2:** RUNX1 in Acute and Chronic Inflammation.

System	Acute Inflammation	Chronic Inflammation	Key Mechanisms	Pathologies
Respiratory	Dual pro-/anti-inflammatory in ALI/ARDS	Sustains ILC2-driven airway inflammation	NF-κB, TGF-β, mitochondrial autophagy	ALI, ARDS, fibrosis, COPD, asthma [36,97,98]
Renal	Promotes tubular NF-κB/IL-6 activation	Persistent fibrosis signaling	NF-κB, IL-6 axis	AKI, CKD, renal fibrosis [35,99,100]
Digestive	JAK/STAT3-mediated protection; Resistin^+^ monocyte expansion	Drives NAFLD, fibrosis via stellate cell activation	JAK/STAT3, NF-κB, TNF	NAFLD, fibrosis, IBD [101,102,103]
Immune/Hematopoietic	Regulates neutrophil/B-cell activation; links hematopoiesis to inflammation	Th1/Th17 skewing, autoimmunity	NF-κB, CD74 axis	Autoimmune disease, RA, leukemia [27,104,105]
Cardiovascular	Upregulated post-MI, remodeling; downregulated in shock	Promotes atherosclerosis, fibroblast activation; protective in aneurysm	NF-κB, TGF-β, STAT3	MI, heart failure, atherosclerosis, aneurysm [23,59,106,107,108]
Metabolic	Modulates acute pancreatitis	Insulin resistance, NAFLD, fibrosis	NF-κB, IL-6, TNF	Diabetes, NAFLD, fibrosis [109,110,111]
Nervous	Neuroprotective in acute injury	NLRP3-driven neurodegeneration	NF-κB, ROS, NLRP3	Alzheimer’s, stroke, neurodegeneration [65,66,112]
Other	—	Chagas (FAK-NF-κB), psoriasis (STAT6/NFATC2), allergic airway inflammation (ILC2)	Multiple axes	Chagas, psoriasis, allergy [113,114,115]

## 4. Conclusions and Future Perspectives

*RUNX1* has transcended its classical role in hematopoiesis to emerge as a pivotal regulator of age-related pathophysiology. Situated at the intersection of inflammatory signaling, epigenetic remodeling, and cellular fate determination, *RUNX1* integrates transient stress responses into durable molecular imprints that perpetuate tissue dysfunction. By coupling “inflammatory memory” with phenotypic fixation, *RUNX1* establishes a self-reinforcing pathological circuit that sustains chronic inflammation, regenerative exhaustion, and functional decline—a mechanism validated across cardiovascular, neural, and hematopoietic systems. Collectively, *RUNX1* represents not merely a participant in aging, but a convergent driver of systemic degeneration.

At the mechanistic level, the regulatory complexity of *RUNX1* remains only partially understood. Isoform-specific roles, lineage-dependent expression patterns, and context-sensitive activities indicate a functional duality—*RUNX1* may facilitate repair under acute injury but exacerbate degeneration under chronic stress. Dissecting this “contextual duality” will require high-resolution approaches such as single-cell multi-omics, spatial transcriptomics, and lineage tracing to chart its spatiotemporal dynamics and identify reversible checkpoints or therapeutic inflection points. At the epigenetic and therapeutic level, *RUNX1* forms an epigenetic “memory module” through cooperation with modifiers including DNMTs, HDACs, and CBFβ, stabilizing senescence-associated transcriptional programs beyond transient stimuli. Decoding this cooperative network could reveal how short-term immune activation evolves into long-term dysfunction and provide new avenues to reverse maladaptive epigenetic imprinting. Although *RUNX1* has long been considered “undruggable”, emerging technologies—including small-molecule interface disruptors, PROTAC-based degraders, and RNA nanotherapeutics—are beginning to challenge this notion. Future strategies should emphasize isoform selectivity, cell-type specificity, and temporal precision to enable a paradigm shift from pathological blockade to functional reprogramming. At the clinical and translational level, *RUNX1* has demonstrated diagnostic and prognostic potential across multicenter studies, particularly in hematologic malignancies and cardiovascular disorders. Dynamic monitoring of *RUNX1* expression may refine risk stratification and therapeutic responsiveness. More importantly, its cross-system involvement in neurodegeneration, vascular remodeling, and immune aging positions *RUNX1* as a unifying molecular target for multi-disease intervention. Integrating *RUNX1* signatures into longitudinal cohorts and interventional trials could accelerate its transition from mechanistic insight to clinical application.

Overall, the study of *RUNX1* marks a conceptual shift in aging biology—from isolated signaling pathways to integrated network regulation. *RUNX1* weaves together inflammation, epigenetic instability, and regenerative failure into a cohesive molecular framework, providing new logic to explain organ decline and systemic aging. Future work should focus on elucidating how *RUNX1* coordinates inter-organ homeostasis and immune adaptation, and on exploring its therapeutic potential in disease intervention, tissue rejuvenation, and healthy aging. Ultimately, *RUNX1* may serve not only as a key node within the aging network, but also as a promising entry point to break the vicious cycle of “inflammation–degeneration”, illuminating a new direction for precision anti-aging strategies.

## Figures and Tables

**Table 1:** *RUNX1* Isoforms: Core Functions and Disease-Related Distinctions.

Isoform	Structural Features	Major Functions	Characteristic Roles in Disease Contexts
*RUNX1a*	Lacks transcriptional activation domain	- Promotes proliferation - Inhibits differentiation	Enhances progenitor expansion and inflammatory sensitivity (e.g., CHIP, myeloid bias)
*RUNX1b/c*	Possesses a complete transcriptional activation domain	- Drives lineage differentiation - Regulates inflammatory gene programs	Promotes chronic inflammation and fibrosis/senescence (cardiac, renal, and age-related tissue pathologies)

## Data Availability

No new data were created or analyzed in this study. Data sharing is not applicable to this article.

## Abbreviations

AKI	Acute Kidney Injury
ALI	Acute Lung Injury
AMPK	5′ AMP-activated protein kinase
ARDS	Acute Respiratory Distress Syndrome
CBFβ	Core-binding factor beta
CKD	Chronic Kidney Disease
COPD	Chronic Obstructive Pulmonary Disease
CXCL1	C-X-C motif chemokine ligand 1
DNMTs	DNA Methyltransferases
eNOS	endothelial Nitric Oxide Synthase
HDACs	Histone Deacetylases
IFN-γ	Interferon gamma
IL-1β	Interleukin 1 beta
IL-6	Interleukin 6
IL-8	Interleukin 8
ILC2	Innate Lymphoid Cell type 2
JAK/STAT	Janus kinase/Signal Transducer and Activator of Transcription
JNK	c-Jun N-terminal kinase
MAPK	Mitogen-activated protein kinase
MMPs	Matrix Metalloproteinases
MyD88	Myeloid differentiation primary response 88
NAFLD	Non-alcoholic fatty liver disease
NF-κB	Nuclear Factor kappa-light-chain-enhancer of activated B cells
NLRP3	NOD-like receptor family pyrin domain containing 3
Nrf2	Nuclear factor erythroid 2–related factor 2
PI3K–Akt	Phosphoinositide 3-kinase—Protein kinase B
PU.1	Transcription factor PU.1
RA	Rheumatoid Arthritis
RHD	Runt homology domain
RNA-seq	RNA sequencing
ROS	Reactive Oxygen Species
RUNX1	Runt-related transcription factor 1
SASP	Senescence-Associated Secretory Phenotype
STAT3	Signal Transducer and Activator of Transcription 3
TGF-β	Transforming Growth Factor beta
TLR3	Toll-like receptor 3
TOLLIP	Toll-interacting protein
TGF-β	Transforming Growth Factor beta
TLR3	Toll-like receptor 3
TOLLIP	Toll-interacting protein
TNF-α	Tumor Necrosis Factor alpha
ULK1	Unc-51-like kinase 1
